# Supplementary material for: A Qualitative Assessment of Clinical Practice Guidelines and Patterns for Congenital Cytomegalovirus in the United States
Source: Int J Neonatal Screen. 2023 Jun 30;9(3):37. doi: 10.3390/ijns9030037 (PMC10366839; doi:10.3390/ijns9030037)
Supplement: Supplementary file 1 [file IJNS-09-00037-s001.zip › IJNS-2362434-supplementary.pdf]

Supplementary Materials

Table S1. Description of codes used to analyze interview transcripts for key themes relating to cCMV clinical practice guidelines and patterns.

Code*	Description
Aware of clinical practice guidelines for cCMV	Tracks awareness of the clinical practice guidelines among participants
Yes, aware of clinical practice guidelines	Affirmative acknowledgment of awareness of clinical practice guidelines by participant
No, not aware of clinical practice guidelines	No acknowledgment of awareness of clinical practice guidelines by participant
Differences between clinical practice guidelines in the United States and internationally	Tracks reference to differences in clinical practice guidelines and patterns, policies, and evidence in the United States and other countries
The use of parallel situations or other diseases/conditions as examples	Tracks diseases, conditions, or recent events that were referred to as parallel to the current situation surrounding cCMV clinical practice guidelines and patterns
Reference to clinical practice guidelines	Tracks references to specific clinical practice guidelines and professional societies
AAP	Participant referred to American Academy of Pediatrics
ACOG	Participant referred to American College of Obstetricians and Gynecologists
ASRM	Participant referred to American Society for Reproductive Medicine
PIDS	Participant referred to Pediatric Infectious Diseases Society
SMFM	Participant referred to Society for Maternal-Fetal Medicine
SOGC	Participant referred to Society of Obstetricians and Gynaecologists of Canada

Importance of patient and family engagement	Tracks references to the importance of including the patient voice
Factors that would mediate change in cCMV clinical practice guidelines and patterns	Tracks reference to factors that would motivate change in cCMV clinical practice guidelines and patterns
Available vaccine	Participant referred to an available CMV vaccine
Evidence of effective treatment or intervention	Participant referred to efforts to provide evidence to support changes in cCMV clinical practice guidelines and patterns
Improved diagnostic testing	Participant referred to a need for improved diagnostics and screening options
New publications and clinical practice guidelines	Participant referred to the impact of new or revised cCMV clinical practice guidelines and patterns
Achievable efforts for near term	Participant referred to immediate actions that could initiate change
Raise awareness	Participant referred to efforts to raise awareness of cCMV
Show evidence of prevention or treatment benefit	Participant referred to efforts to provide evidence for a new clinical practice guideline option
Ongoing efforts to increase awareness	Tracks references to existing efforts in the field to educate and increase cCMV awareness among healthcare providers and patients
Reasons for current clinical practice patterns	Tracks specific reasons participants noted as reasons that current patterns are the way they are
Competing priority for OB/GYNs	Participant referred to cCMV being a lower priority compared with other concerns
Lack of relevance to obstetric care	Participant indicated cCMV lacks relevance to obstetric care
Low awareness of CMV	Participant referred to low awareness of cCMV
No evidence to change practices	Participant referred to a lack of evidence to change clinical practice
No treatment or intervention	Participant referred to the lack of available options for treatment or interventions

Not cost-effective to screen	Participant referred to the cost of universal screening
Unreliable diagnostic options	Participant referred to a lack of appropriate and trusted screening practices
Develop data to highlight problem and potential solutions	Participant referred to the need for evidence generation
Development of treatment and vaccines	Participant referred to vaccine development
Educate patients	Participant referred to raising awareness of cCMV among patients
Educate providers	Participant referred to cCMV education for providers
Increase awareness	Participant referred to increase overall awareness of cCMV among the public and providers
Role of the US pregnancy termination policy	Tracks references to participants' perception of how the termination policy in the United States impact decisions made by OB/GYNs relative to policies in other countries
Impactful for OB/GYNs	Participant indicated domestic pregnancy termination policies impacting decision-making among OB/GYNs
Not impactful for OB/GYNs	Participant indicated domestic pregnancy termination policies do not impact decision-making among OB/GYNs

*Codes were organized hierarchically in a parent-child relationship to derive high-level and specific themes. Codes representing high level themes are presented in gray rows, with corresponding specific themes below in white rows.

AAP, American Academy of Pediatrics; ACOG, American College of Obstetricians and Gynecologists; ASRM, American Society for Reproductive Medicine; cCMV, congenital cytomegalovirus; CMV, cytomegalovirus; OB/GYN, obstetrician/gynecologist; PIDS, Pediatric Infectious Diseases Society; SMFM, Society for Maternal-Fetal Medicine; SOGC, Society of Obstetricians and Gynaecologists of Canada.

Table S2. Selected excerpts adapted from interviews in which participants discussed the differences in cCMV clinical practice guidelines and patterns, policies, and evidence between the United States and other countries.

**Interview transcript excerpt**
The argument that there is nothing to offer is incorrect, not the right argument during pregnancy. Down syndrome screening is offered in the absence of treatment, so the argument that providers should not discuss CMV with their patients because there is no treatment is not logical. Prenatal diagnostics such as an amniocentesis to check whether the baby is infected can be offered.
A problem in the US is that many women are seen slightly later in their second trimester, raising a problem of access. If CMV screening is performed, it should ideally be in the first trimester to capture early primary infection. However, it is not clear if screening in the first trimester is acceptable in the US.
The interviewer noted that most women in the US are screened or have an ultrasound at least once in the early second trimester, but in reality most women have multiple ultrasounds even in the first trimester. This does not seem to be an apparent challenge in the US
In Quebec, it is standard for all pregnant women to be given time off from work during pregnancy.
Internationally, guidelines are variable in addition to there being practices including maternal CMV screening and education counseling that are outside of guideline recommendations. For example, in Israel counseling and screening practices are evolving due to a legal case. [The interviewer] asked if there would be results from international practice patterns that could influence the US or is it just the data?
This is dependent upon the area of medicine. In cardio-obstetrics, there is not much strong data available, so the recommendations used are based on little data. But in the field of CMV, American medicine can be 'haughty' and may not be willing to follow international evidence. Although, if the evidence and recommendations are broadly adopted in European nations, this may pressure the US medical field to do the same. But this is not yet demonstrated.
In Australia and New Zealand, scientists in positions of authority showed an interest in CMV and had been able to push for recommendations on CMV counseling and sharing information with patients. A similar shift has occurred in Canada and had allowed them to really jump ahead. CMV screening with dried blood spots was incorporated as part of Canada's universal newborn screening program. Although infection is better detected by saliva or urine testing, not all cases will be identified, but it is a step forward.
European CMV meetings involved more OB/GYNs than they currently do in the US.
Regarding stakeholder groups that could be engaged, international CMV organizations have targeted working with midwives. While not a main focus in US, midwifery is trending up sothis may be something that organizations could explore.
In the US, hearing loss is referred to as a sequala while Europeans refer to it as a symptom. This is a difference in definition and perception between the US and other nations. The Europeans are more correct here as CMV is a spectrum, not a binary infection, either symptomatic or asymptomatic. On one end of the spectrum, there are asymptomatic patients that have no symptoms at all, then there are asymptomatic cases with hearing loss, symptomatic cases that present with transient symptoms, but no complications related to CNS function, all the way to symptomatic with symptoms and severe CNS complications.

The understanding is that ACOG might be going back to take another look as there has been recognition that Canadian guidelines are more up to date and embracing of care for patients with CMV, so there is a need to look into this.
The challenge they face is figuring out the treatment and recommended use of antivirals in newborns. Criteria are currently at odds, especially around the issue of hearing loss. These differences are stark between Canadian, U.S., and European/Israel recommendations around treatment.
Other countries are far more advanced than the US For example, in Israel screening is performed every trimester and there is no limit on termination meaning there are more options for consideration. Italy and France are much more research oriented that the US and have led studies for CMV treatment and prevention.
It would be interesting to look at the efforts made in other countries. Israel's screening methods are well-established, Canadian guidelines are clearly important, but guidelines from France, UK, Italy, and Australia may be also important to consider because they could motivate practice advancement.
A possible reason why the US is hesitant to leverage current evidence is that it is foreign data, and we have a tendency to be skeptical and elitist in this regard. The US is conservative regarding uptake and implementation until the data has been published by US investigators; however, this is not true of the converse.
Data around preventative behaviors were not great but were still compelling and it would be worthwhile to provide that to patients and families. Families are highly accepting of information on preventative measures to lower the risk of CMV transmission when informed of the risk and potential sequelae. At the very least, we could do a better job of conveying the risks and preventative measures to the families.
When families are aware of CMV screening, they want to be screened. This increased demand for screening could in itself facilitate greater awareness in the community and for more universal screening options.

ACOG, American College of Obstetricians and Gynecologists; cCMV, congenital cytomegalovirus; CMV, cytomegalovirus; CNS, central nervous system; OB/GYN, obstetrician/gynecologist.

Table S3. Selected excerpts adapted from interviews in which participants discussed factors underlying current cCMV practice patterns. Excerpts are categorized by their specific themes coded in interviews.

**Specific theme**	**Interview transcript excerpt**
Competing priority for OB/GYNs	CMV is a competing priority for OB/GYNs due at least in part to the number of issues they contend with, such as other viral conditions with when looking at high-risk pregnancies.
	There is so much for OB/GYNs to discuss and many concerns that they cannot do anything about. Not all possible negative outcomes for the infant are addressed. The best thing OB/GYNS can do is monitor good health and hygiene in the absence of testing or treatment for CMV.
Lack of relevance to obstetric care	As obstetricians do not see the sequelae, it is possible that CMV has less relevance.
	Shared the anecdote about a pregnant woman who died of H1N1. The documentation showed the obstetrician never offered the vaccine. So, it is relevant to say OB/GYNs should counsel patients based on guidelines, but this should be done in the preventive care rather than the obstetrics space
	For OB/GYNs on the ground, there are subtle steps that create confusion and struggles. The question is, if all women are at risk, why should they screen for CMV? Irrespective of the outcome, the same advice would be given to the patient.
Low awareness of CMV	With the CMV legislation passed in 2013, one would expect a fair degree of awareness among the community. There is some level of awareness among primary care and physicians, especially on the pediatric side, but awareness among obstetricians and the general public is still relatively low.
	There is an ongoing study in which women are randomized into an video education program. The experiment is a repeat of one performed in 2013 to look at seroconversion, reinfection and shedding in mothers. Some women received CMV disease awareness education and some received stress reduction programing. Participants were followed up after 12 weeks for serology samples. The population was very urban with many people of color. In the 2013 study, only 15% of women had heard of CMV. In the current study of 180 women, only 8% have heard of CMV.
	Maternal fetal medicine specialists are thought of as experts but the education on CMV is lacking, and, in many cases, comes from the schooling received and not clinical practice.
No evidence to change practices	OB/GYNs may feel that they are not able to change practices unless told to do so; they are largely dependent on the guidelines to initiate change in practice. Asking OB/GYNs to change practices at this stage is asking them to do research and change practices based on evidence, but guidelines are required to dictate practice.

	Diagnostic approaches vary from testing urine to blood samples, and screening varies from recommendations for universal, to screening only symptomatic infants, to targeted newborn screening. Many health departments are interested in creating awareness of universal screening; however there is no consensus as to whether it will be beneficial or not, or whether such screening will create more anxiety for pregnant people.
	There is no impetus for change without an evidence-based reason. Everyone would likely be very supportive and excited about something to aid in CMV prevention.
	Guidelines come from the evidence, cost benefit analyses, and the availability of treatment opportunities. There is no evidence for a treatment; this lack of evidence severely impacts the costs and benefits weighing.
	Evidence to change practices may come in the form of a treatment or a vaccine.
	ACOG's stance is that there is no evidence that indicates talking about prevention with patients decreases infection.
No treatment or intervention	The absence of an easy intervention underlies current clinical practices.
	The interviewer noted that they had heard that OB/GYNs are in fact not mentioning CMV because they do not have treatment to offer, but this is paternalistic (makes the decision for the patient without giving them the chance to elect to know more). Information should be offered because knowledge is empowering.
	The participant agreed on this. CMV will always be a difficult disease to deal with, but knowledge is helpful.
	A classic teaching of cost effectiveness is that you only screen for something if there is a treatment or prevention option available. For now, in the case of CMV and the absence of treatment or prevention, doing anything more than counseling patients about proper hygiene just raises anxiety and concern.
	If there is no vaccine available, it would not be clear what the conversation with patients would be about. It would just be about general awareness, but the list of what needs to be discussed with patients is already extensive. We should really only talk about a disease if there is something we can do about it.
	Talking about CMV will not accomplish anything. It would raise awareness, but patients would be left with worry because there is nothing they can do to prevent it. Providers are not against mentioning CMV to patients, they just do not have anything to do about it. Only general hygiene counseling should be given.
	A problem with screening is deciding what will be done with the results and how to appropriately advise the patient. Many physicians do not feel comfortable explaining the result.
	The guidelines exist as they are because there is no cure. Why go looking for something there is no cure for? What motivation would ACOG have for recommending screening for something without a treatment approach?

	CMV is the most prevalent infectious disease, there is no other reason to not screen for it. There is no treatment and that is the reason for not screening. Hepatitis C, for example, is a disease that is moving towards universal maternal/fetal screening now that there are treatment options. The stance may be taken by ACOG may be to avoid creating anxiety in patients without a reason or treatment.
	Guidelines are based on evidence, cost benefit analysis, and treatment opportunities. There is no evidence for a treatment and this severely impacts the costs and benefits weighing.
Not cost effective to screen	A classic teaching of cost effectiveness is that screening is only performed if there is a treatment or prevention option available. In the case of CMV, in the absence of treatment and prevention doing anything more than educating patients about proper hygiene just raises anxiety and concern.
	Without prevention of treatment, screening is not cost effective, nor does it make sense in the patient's eyes. Screening would only generate more worry in the absence of something to do about the results.
	Guidelines are based on evidence, cost benefit analysis, and treatment opportunities. There is no evidence for a treatment and this severely impacts the costs and benefits weighing.
Unreliable diagnostic options	The obstetrics department at the University of Utah is collaborating on a project looking at novel diagnostics which, if they come to fruition, may provide greater clarity. The diagnosis of secondary infection is problematic, so it is a challenge to identify infants with prenatal CMV infection.
	Diagnostic options are not great. They are not predictive, specific, or reliable. Such ambiguity is a challenge for OB/GYNs to deal with in the CMV disease space.
	In certain areas, there is no access to avidity testing. Avidity is a way to ensure the CMV infection is recent or not based on avidity of IgG. In certain Canadian provinces, avidity testing is available, but not all. This may be a problem in the US too if avidity testing is available if some regions.
	The asymptomatic patients are a great unknown. Symptomatic patients present clinically, if caught, but there is not much knowledge regarding patients who are not identified because they are asymptomatic. This is a gap in evidence. While not a large number, asymptomatic cases are still present. It is important to distinguish patients who are truly healthy and will not experience long-term sequelae from the symptomatic and asymptomatic cases.
	A study in Brazil (Marisa Musi Pena) reported a 97% seropositivity when assessing shedding and seroconversion/reinfection. There is no clean definition of what reinfection is. Women shed differently in different compartments, and typically in low levels (unlike with a baby) so the focus should be on immunological principles. Shedding is highly intermittent, making it difficult to accurately measure by PCR.

	One of the challenges today is the question of 'what are we screening for'? Of course, we are screening for cCMV but are we screening for newborns at risk for hearing, speech and development concerns (Ontario Eddy program), or are we screening all newborns because there is the potential for all of them to develop sequelae down the road? The evidence suggests the latter because there no evidence that the 85% of asymptomatic cases should not be screened. This is a big issue that needs to be resolved somehow.

ACOG, American College of Obstetricians and Gynecologists; cCMV, congenital cytomegalovirus; CMV, cytomegalovirus; CNS, central nervous system; IgG, immunoglobulin G; MFM, maternal-fetal medicine; OB/GYN, obstetrician/gynecologist; PCR, polymerase chain reaction.

Table S4. Factors identified as contributing to cCMV clinical practice guidelines and patterns stratified by participant profession.

Theme (code)	Description of code	Participant profession	n (%)
Factors contributing to practice patterns	Indicates reference to any contributing factor	Total (n = 8)	7 (87.5%)
		Pediatrician (n = 4)	4 (100%)
		MFM (n = 2)	2 (100%)
		OB/GYN (n = 2)	2 (100%)
Competing priority for OB/GYNs	Indicates reference to CMV/cCMV being a lower priority compared with other concerns	Total (n = 8)	2 (25%)
		Pediatrician (n = 4)	1 (25%)
		MFM (n = 2)	1 (50%)
		OB/GYN (n = 2)	0 (0%)
Lack of relevance to obstetric care	Indicates reference to CMV/cCMV as not relevant	Total (n = 8)	3 (37.5%)
		Pediatrician (n = 4)	2 (50%)
		MFM (n = 2)	1 (50%)
		OB/GYN (n = 2)	0 (0%)
Low awareness of CMV	Indicates reference to low awareness of CMV/cCMV	Total (n = 8)	2 (25%)
		Pediatrician (n = 4)	2 (100%)
		MFM (n = 2)	0 (0%)
		OB/GYN (n = 2)	0 (0%)
No evidence for change	Indicates reference to a lack of evidence to support change	Total (n = 8)	5 (62.5%)
		Pediatrician (n = 4)	3 (75%)
		MFM (n = 2)	2 (100%)
		OB/GYN (n = 2)	0 (0%)
No treatment or intervention	Indicates reference to the lack of available options for treatment or interventions	Total (n = 8)	5 (62.5%)
		Pediatrician (n = 4)	2 (50%)
		MFM (n = 2)	2 (100%)
		OB/GYN (n = 2)	1 (50%)
Not cost-effective to screen		Total (n = 8)	3 (37.5%)

	Indicates reference to cost ineffectiveness of universal screening	Pediatrician (n = 4)	1 (25%)
		MFM (n = 2)	2 (100%)
		OB/GYN (n = 2)	0 (0%)
Unreliable diagnostic options	Indicates reference to a lack of appropriate, reliable, or trusted screening practices	Total (n = 8)	5 (62.5%)
		Pediatrician (n = 4)	4 (100%)
		MFM (n = 2)	0 (0%)
		OB/GYN (n = 2)	1 (50%)

n, number of participants referring to the specific theme of interest. Specific themes are presented in rows below the parent theme in the gray row.

cCMV, congenital cytomegalovirus; CMV, cytomegalovirus; MFM, maternal-fetal medicine; OB/GYN, obstetrician/gynecologist.

Table S5. Selected excerpts adapted from interviews in which participants discussed the importance of raising awareness of cCMV and how this may mediate change in clinical practice guidelines and patterns in the United States.

**Interview transcript excerpt**
To reach the younger generation, social media and applications should be included as part of education and engagement efforts.
Education of OB/GYNs will be essential for shifting guidelines and practices.
OB/GYNs and pediatricians should be involved in messaging about CMV treatments.
ABOG could mandate CME includes articles on CMV to ensure more extensive reach.
Patient advocacy groups would be very influential in moving the needle. This is a constant seen in the cardio-obstetric realm: patient voice is included in decision-making and recommendation development. Including patient voice of affected persons who had CMV and found out after the fact should be included in the narrative.
Large professional societies are finally asking patients to be at the table, which is important for decision-making.
75% of general OB/GYNs obtain information from CME or consultations from specialists. Unless required, OB/GYNs do not have time to conduct the research. As continuous education is required, ABOG mandates may be impactful for influencing the adoption of new practice patterns.
There are people with knowledge regarding the effective messaging of information and how to tackle complex issues like this. It will be crucial to determine how to move the needle on the number of women who know about CMV.
MFM specialists have influence in this regard, but many rely on their knowledge from medical school, which may be out of date.
AAP is the major thought leader or driver on the newborn side; the conversation of raising awareness to mediate change in guidelines and patterns can be continued at their upcoming conference.
Pediatric hospitalist groups should be included in the discussion because of their proximity to newborn care and their established and potential roles in targeted screening.
For example, there was a patient who had twins with cCMV, one of whom has passed away. The patient started a parental group that is very active in raising CMV awareness.
The CDC is important as they have been impactful in shifting positions in Hepatitis C in pregnancy. Because the CDC and California Public Health Department put their weight behind Hepatitis C, there was a shift in guidance to screen all patients in the last few years.
ACOG releases opinions and the practice guidelines and physicians are required to review them for annual board recertification. True clinical physicians have limited time to review studies, but ACOG distributes practice bulletins that are expected to be followed by all OB/GYNs that are part of ACOG.

MMWR and the CDC may be standard educators/disseminators of information on CMV.
There are also political advocacy groups that aim to have screening as a requirement in states across the country. These organizations would be another way of disseminating CMV information.

AAP, American Academy of Pediatrics; ABOG, American Board of Obstetrics and Gynecology; ACOG, American College of Obstetricians and Gynecologists; cCMV, congenital cytomegalovirus; CDC, Centers for Disease Control and Prevention; CME, continued medical education; CMV, cytomegalovirus; MFM, maternal-fetal medicine; MMWR, Morbidity and Mortality Weekly Report; OB/GYN, obstetricians/gynecologist.

Table S6. Selected excerpts adapted from interviews in which participants discussed the importance of patient engagement and voice in evolving cCMV clinical practice guidelines and patterns in the United States.

**Interview transcript excerpt**
Mothers of infants with cCMV infection can feel betrayed that they were not informed about CMV beforehand.
There is some negative feedback on receiving "more information of scary viruses" but overall many people feel that knowledge is power and want to understand the risks and the ambiguity of CMV infection and disease.
Without having been provided with the information or education, patients feel like they were betrayed. They feel that maybe they could have done something different.
OB/GYNs are not mentioning CMV because there is no treatment to offer, but this is paternalistic (makes the decision for the patient without giving them the chance to elect to know more). Information should be offered because knowledge is empowering.
Although dealing with CMV disease will always be difficult, knowledge is helpful.
OB/GYNs should take part in the messaging regarding health maintenance and education, including pre-pregnancy counseling. OB/GYNs should be thinking of CMV as a topic to discuss with their patients. While it is possible, but unlikely, that OB/GYNs could be vaccinators they should still discuss CMV with patients in a counseling role.
A recent study was conducted to determine whether women want to know the risk of CMV. It was found that if patients are counseled about CMV, they want to know their risk. The idea of not wanting to review CMV risk with the patient is a paternalistic approach.
[Patient associations were discussed including the National CMV foundation and a Canadian CMV Association. Such associations are very vocal and bring the patient voice to the table so working with patient associations is a good way to incorporate the patient voice around CMV. If physicians are pressured by their patients to discuss CMV, providers will start talking about it more. It would be great if these discussions were driven from the patient's side.
The lack of patient voice is something that should change in the future; patient partners should be involved in the development of guideline documents.
Knowledge is power. When they have the information, patients get to decide what to do about it and that avoids the paternalistic relationship between patient and provider.
The current recommendations have taken away choice and shared decision-making, which makes some doctors uncomfortable. If patients are aware, they get to choose.
Parent perspectives are very important to allow patients to feel they can make their own decisions. Patients deserve to know the risks and what measures they can implement to protect themselves. Many people feel it is patronizing of professionals to avoid educating patients about CMV.
It is important to look at this language from the patient's lens and what they may want to know to help this conversation along. They do not want to appear as if trying to drive change in the guidelines but rather empowering patients and providers to act.

cCMV, congenital cytomegalovirus; CMV, cytomegalovirus; OB/GYN, obstetrician/gynecologist.
